# Impact of COVID‐19 on a worksite weight loss program for employees with overweight and obesity

**DOI:** 10.1002/osp4.653

**Published:** 2022-12-21

**Authors:** Che Young Lee, Michael C. Robertson, Kendahl Servino, Thuan Le, Margaret Raber, Katherine Oestman, Karen M. Basen‐Engquist

**Affiliations:** ^1^ Department of Behavioral Science The University of Texas MD Anderson Cancer Center Houston TX USA; ^2^ Department of Health Disparities Research The University of Texas MD Anderson Cancer Center Houston TX USA; ^3^ Department of Nutrition, Metabolism & Rehabilitation Sciences The University of Texas Medical Branch at Galveston Galveston TX USA; ^4^ University of Nevada Reno School of Medicine Reno NV USA; ^5^ USDA / ARS Children’s Nutrition Research Center Department of Pediatrics‐Nutrition Baylor College of Medicine Houston TX USA; ^6^ Be Well Communities Cancer Prevention and Control Platform The University of Texas MD Anderson Cancer Center Texas USA

**Keywords:** COVID‐19 pandemic, health behaviors, weight management, digital weight loss program

## Abstract

**Objective:**

The COVID‐19 pandemic has been shown to be negatively associated with physical activity engagement, adherence to healthy diet, and weight management among people with obesity. The current study examined COVID‐19‐related changes in weight, physical activity (PA), and diet among employees with obesity or overweight who participated in Vibrant Lives (VL), a worksite weight loss program.

**Methods:**

School district employees participated in the 6‐month VL weight loss program and were categorized into non‐COVID‐era participants and COVID‐era participants. Participants completed questionnaires about PA and dietary intake at baseline and follow‐up. COVID‐era participants reported the effects of pandemic on their behaviors. Changes in weight, PA, and diet were compared between groups using multilevel linear mixed models and logistic regression models.

**Results:**

A total of 266 participants (non‐COVID, n=173; COVID, n=93) were included. Significant weight loss (non‐COVID, −2.3 kg vs. COVID, −1.3 kg) and increases in moderate‐to‐vigorous PA minutes (non‐COVID, 48.7 min vs. COVID, 61.5 min) were observed associated with the program, but no significant differences in changes between the groups were found. Compared to non‐COVID participants, COVID participants decreased fast food consumption (*P*=.008) and increased sugar‐sweetened beverage intake (*P*=.016). Higher frequency of snacking and overeating were reported as barriers to a healthy diet.

**Conclusion:**

The COVID‐19 pandemic was negatively associated with healthful dietary behaviors. The information obtained from participants regarding the reasons for their pandemic‐related changes in diet may help identify strategies to encourage healthier behaviors and weight management among people who have been negatively affected by the COVID‐19 pandemic.

This article is protected by copyright. All rights reserved.

## Introduction

Worksite weight loss intervention programs have the potential to decrease the prevalence of obesity and obesity-related comorbidities among employees (1-4). Worksites are ideal settings in which to implement health-promotion programs, in part because they commonly include robust communication and support systems for employees (5). Worksite-based educational programs that offer behavioral strategies for promoting physical activity (PA) and healthier diets have helped employees lose weight (6, 7) and improve their diet and levels of PA (8, 9). Programs encouraging behavioral changes may help employees manage their weight and avoid regaining lost pounds.

After COVID-19's emergence, many governments imposed stay-at-home orders (lockdowns) to limit the spread of infection. During this time, workplaces were closed and many people were restricted to engaging in only outdoor activities with social distancing or staying at home altogether (10). Working from home may disrupt daily routines; for example, employees may decrease their occupational PA and increase their time spent in front of a computer or television (screen time). They may also lower adherence to healthy diet practices; some may increase their snacking and caloric intake owing to increased screen time (11) and boredom from activity limitations (12). Indeed, people reported more snacking and overeating during the COVID-19 lockdown (13). Moreover, people gained weight at a rate of 1.5 kg per month during the stay-at-home-order period (14).

Among individuals with obesity, COVID-19–related barriers to engaging in PA and healthy eating behaviors may prevent weight loss. Previous studies highlighted the challenges adults with obesity have faced regarding weight gain and adherence to healthy behaviors for

This article is protected by copyright. All rights reserved.

weight loss since the outbreak of the pandemic (15). In particular, individuals with obesity have reported difficulty achieving their weight loss goals (16, 17), and weight loss program participants have shown a decrease in short-term weight loss and food intake self-monitoring (18) during the pandemic. Worksite weight loss programs for employees with overweight and obesity may have likewise been affected by COVID-19; working from home, exercise facility closure, and difficulty in following a healthy diet may have decreased the efficacy of such programs.

To facilitate employers' providing effective weight loss programs for employees with obesity or overweight, there is a need to explore how COVID-19 has affected healthy behaviors and to identify the barriers to weight loss, engagement in PA, following a healthy diet, and the implementation of worksite weight loss programs during the pandemic. The purpose of this study was to examine the effect of COVID-19 and the pandemic shutdown on weight and behaviors of school district employees who participated in Vibrant Lives (VL), a multi-year digital worksite weight loss program. Previous VL study using the objectively measured physical activity data (Fitbit) from this program found that study participant during the 2019-2020 school year had a greater increase in vigorous physical activity than those who participated in the previous year, despite a greater reduction in overall activity (19). Here COVID-19 related effects on weight, dietary behavior, and self-reported physical activity were reported. It is hypothesized that, compared to people participating in VL before the pandemic, those participating during the pandemic would be less likely to engage in PA program dietary/eating behavior recommendations, and lose weight.

**Methods**

This article is protected by copyright. All rights reserved.

This study was a secondary analysis of data from the VL program. The VL program is a digital weight loss program focused on behavioral changes, such as increasing PA and developing healthy eating habits using the adapted Diabetes Prevention Program (20). The VL program was offered during 3 school years, 2017-2018 (year 1), 2018-2019 (year 2), and 2019-2020 (year 3).

*Participants and intervention*

Participants in the VL program were employees of the Pasadena Independent School District in southeast Texas. Eligible participants had a body mass index (BMI) greater than 25 (in year 1) and greater than 27 (in years 2 and 3), signed a medical release form, and completed a baseline survey. The enrollment started in late September/early October of each school year with the intervention starting in November. Participants were divided into 2 groups based on the school year in which they participated: non-COVID (2017-2019 school years) and COVID (2019-2020 school year). The study was approved by the Institutional Review Board of The University of Texas MD Anderson Cancer Center, and all participants gave their written informed consent before data collection.

All participants enrolled in the 6-month VL program were educated about healthy eating behaviors and PA and were taught skills to support their efforts. The program included materials adapted from the Diabetes Prevention Program, which were weekly sent to participants through email about tips for being physically active, reducing calorie intake and healthy choice, and weight loss. The behavioral goals emphasized in the DPP included 150 minutes of moderate to vigorous intensity activity or 10,000 steps per day, a calorie goal of

Accepted Article

This article is protected by copyright. All rights reserved.

1200-2000 calories per day and a fat gram goal of 33-55 grams per day, depending on starting weight. Participants received text messages with tips for behavior changes, Fitbit activity monitors, and Wi-fi–connected scales (Fitbit Aria Air Smart Scale, San Francisco, CA). Participants were encouraged to use the Fitbit mobile app to monitor dietary intake and physical activity. They also participated in challenges each month from November – April in which they competed for prizes if they met the challenge objectives. Challenges included the "No gain challenge" (no weight gain from Thanksgiving to New Year's Day), the Heart Health Challenge to increase active minutes (as identified by the Fitbit) by 15%. In the 2018-2019 and 2019-2020 participants had the opportunity to engage with a private Facebook group in which weekly challenges were posted, e.g. try a new vegetable, scavenger hunt, and participants were encouraged to post pictures related to the challenge.

*Measures*

Participants completed a survey that asked for basic demographic information such as age, race, sex, education level, and marital status. Weight was measured using Fitbit Aria Smart Scale at baseline and after the program.

Self-reported PA and dietary intake were assessed using items from the Health Information National Trends Survey (PA and fruit and vegetable intake) (21) and the Health of Houston Survey (other dietary intake questions) (22). The questions for PA and dietary intake were listed in the Table 1. For aerobic PA, a total minutes of moderate-to-vigorous physical activity (MVPA) during the week were calculated by multiplying the number of days per week participants did leisure-time MVPA and the typical length of these sessions. A dichotomous

This article is protected by copyright. All rights reserved.

variable signifying whether the participant met recommendations was calculated based on whether he/she did at least 150 minutes of MVPA and 2 sessions of muscle-strengthening exercise per week was calculated. For dietary intake, responses were recorded according to whether participants 1) consumed red meat (≤ 2 times/day, score = 1; ≥ 3 times/day, score = 0); consumed fruit (≥ 1 cup/day, score = 1; < 1 cup/day, score = 0); consumed vegetables (≥ 3 times/day, score = 1; <3 times/day, score = 0); consumed fast food ((≤ 2 times/week, score = 1; ≥ 3 times/week, score = 0); ate breakfast (every day in the past week, score = 1; 0-6 days in the past week, score = 0); consumed sugar-sweetened beverages (never in the past week, score = 1; at least once in the past week, score = 0); and read the nutritional information on a menu (often or always, score = 1; never or sometimes, score = 0).

For COVID-19 participants only, a COVID-19 questionnaire was added at follow-up to ask whether the COVID participants had changed their behaviors because of the pandemic. In this study, participants reported whether their food quality, snacking frequency, overeating, meal-cooking frequency, meal-cooking variety, and alcohol consumption had 1) decreased, 2) not changed, and 3) increased. If applicable, participants also selected the reason(s) that their dietary intake had been affected by COVID-19.

*Statistical analyses*

Descriptive data at baseline were analyzed for participants who completed and dropped out as well as in the non-COVID and COVID groups. One-way analysis of variance tests were used for continuous variables (age and BMI), and Pearson's chi-squared tests were used for categorical variables (i.e., race, sex, education). Multilevel repeated-measure linear mixed

This article is protected by copyright. All rights reserved.

models (for continuous variables) and logistic regression models (for dichotomous variables) were used to assess changes in weight, PA, and dietary intake between baseline and follow-up (time) among the non-COVID and COVID groups (group) and the interaction between time and group (time*group). Participants were nested within schools in the analyses. All statistical analyses were performed using STATA statistical software, version 15.1 (StataCorp LP, College Station, TX) and unstandardized beta values (β), odds ratios (ORs) with standard errors (SEs), and *P*-values were reported. A *P*-value of .05 was considered statistically significant.

**Results**

Table 2 shows demographic data retrieved at baseline for the all VL participants (n=380). Completers are based on participants who completed follow-up assessments, while drop-outs are based on those who did not complete follow-up assessments. Drop-out rates of non-COVID and COVID groups were 27% (n=64) and 35% (n=50), respectively, which were not significantly different (χ=2.692, P=.101). No statistical differences of baseline characteristics between completers and drop-outs were observed. The analyses included 266 participants who completed baseline and follow-up assessments. The participants' baseline characteristics by group are reported in Table 3. There were no statistical differences between the non-COVID and COVID participants at baseline. Differences in outcomes between the 2017-2018 and 2018-2019 (non-COVID) school years were tested; as there were no significant group by time interaction effects, the data from these two years were combined (data not shown).

Table 4 shows weight, PA level, and dietary variables of the non-COVID and COVID groups at baseline and follow-up. Significant weight loss were observed (non-COVID, −2.3 kg vs.

This article is protected by copyright. All rights reserved.

COVID, −1.3 kg; β=-2.35; SE=0.35; *P*<.001) and increases in MVPA minutes (non-COVID, 48.7 min vs. COVID, 61.5 min; β=49.10; SE=11.63; *P*<.001) in each group, but no significant differences in the changes between the groups (weight, β=1.08; SE=0.59; *P*=.068; MVPA, β=12.40; SE=19.14; *P*=.517). Non-COVID group showed more clinically significant weight loss (5%) compared to the COVID group (21.3% vs. 13.9%; Pearson chi-square test; *P* = .048). Participants in both groups reported significant reduction in red meat (≤ 2 times/week; OR=1.49; SE=0.25; *P*=.019) and fast food consumptions (≤ 2 times/week; OR=2.08; SE=0.35; *P*<.001), increases in consumptions of fruit (≥ 1 cup/day; OR=2.14; SE=0.38; *P*<.001) and vegetables (≥ 3 cups/day; OR=1.71; SE=0.38; *P*=.016), and use of menu calorie information to choose foods (always/often; OR=3.08; SE=0.56; *P*<.001) after the VL program. Compared to the non-COVID group, the COVID group were more likely to limit their fast food consumption (≤ 2 times/week; OR=2.19; SE=0.65; *P*=.008) but less likely to limit their sugar-sweetened beverage consumption (never in the past week; OR=0.95; SE=0.54; *P*=.016) after the program.

Participants in the COVID group also reported changes in eating behavior related to the COVID-19 pandemic and the reasons for these changes. According to the responses, 66% of participants reported an increase in snacking frequency, 83% reported meal cooking more frequently, and 47% reported an increase in overeating, behaviors which can affect diet quality and weight loss during the pandemic (Fig. 1).

**Discussion**

The current study examined how the COVID-19 pandemic affected changes in weight, PA, and dietary intake among school district employees with overweight and obesity who

This article is protected by copyright. All rights reserved.

Accepted Article participated in a 6-month digital weight loss program. Other studies have shown the negative effects of COVID-19 on healthy behaviors and weight, including difficulty losing weight, decreased PA participation, and difficulty maintaining a healthy diet (15-17, 23). In particular, adults with obesity gained weight during the pandemic (15, 16, 23), decreased PA (16), and had unhealthy diet habits (16, 17). In terms of physical activity, most dietary behaviors and weight loss, our data showed improvement among school employees with overweight and obesity who participated in the VL weight loss program in both the COVID and the non-COVID groups. The mean weight loss was lower for participants in the COVID-19 year than for those in the non-COVID-19 year, but the difference between the two groups was not statistically significant. However, our data showed that clinically significant weight loss (5%) occurred more often in the non-COVID group than in the COVID group (24). Likewise, in a recent study, significantly less weight loss was observed among the COVID-19 cohort (2020) compared to the control cohort (2019) (18). Given that clinical weight loss may be particularly important for people with overweight and obesity for preventing comorbidities, the fact that the COVID-19 pandemic has been shown to be negatively associated with desirable weight loss and health outcomes is especially concerning from a public health perspective (23).

Interestingly, this found that the COVID group showed a greater increase in the mean number of weekly self-reported MVPA minutes and in the percentage of participants meeting MVPA and strengthening exercise recommendations, although these group differences were not statistically significant. This is consistent with our previous paper showing higher "very active minutes" as recorded by participants' Fitbits (19). Previous studies yielded similar results, including an increase in self-reported exercise on weekdays (25) and an increase in

Accepted Article

This article is protected by copyright. All rights reserved.

MVPA minutes (19) during the COVID-19 lockdown. However, most previous studies have shown decreases in MVPA related to the COVID-19 lockdown (26-29). More than half of the COVID group participants in our study reported that their aerobic MVPA and strengthening exercises either had not changed, remained the same amount of PA as before the pandemic but that they had substituted different activities, or increased overall. Participants reported that the pandemic had led them to try to get fitter to protect their health, find new resources for engaging in PA at home, and have more time and motivation to exercise (19). A recent study found that patients who received telemedicine obesity care were about 2.5 times more likely to lose weight and increase exercise participation compared to patients who did not receive telemedicine care during the COVID-19–related lockdown (30). Internet-based, digital, or telemedicine interventions may have the advantage of not being affected by the pandemic lockdown. It is possible that involvement in the Vibrant Lives weight loss program, in which evidence-based content was delivered virtually and thus not interrupted by the COVID shutdown, helped to buffer participants against COVID-related reductions in physical activity less healthful eating patterns.

Nevertheless, many Vibrant Lives participants in the COVID group reported struggles in following a healthy diet during lockdown. Consistent with the results of previous studies (26, 28, 30-33), these results showed a pandemic-related increase in snacking frequency and overeating among participants. In particular, those in the COVID group significantly increased their consumption of sugar-sweetened beverages after the intervention. Participants may have increased their snacking and overeating during the pandemic for the same reasons that they reported changing their overall eating behaviors such as that they had more time for cooking

This article is protected by copyright. All rights reserved.

and baking sweets, more frequent stress-induced eating, more difficulty resisting snacking at home, and a higher likelihood of eating because they were bored. However, participants in the COVID-19 group showed significantly less fast food consumption than those in the non-COVID group. A similar result was found in a previous study (28), indicating that people were less likely to dine at or pick up fast food restaurants during lockdown. Although our results regarding PA related to the pandemic were mixed, most relationships of COVID-19 with diet and eating behavior were negative, indicating that the pandemic put up more barriers to adherence to a healthy diet. These barriers may be affected by other factors, such as stress and mood (33, 34). More research is required to identify ways to overcome the pandemic-related barriers to following a healthy diet and managing stress.

Our study had some limitations that could affect interpretations of its results. The changes of PA and dietary intake were subjectively assessed using surveys, which have well-documented limitations. On the other hand, a strength of our study was its use of a participant survey, the results of which could be used to determine whether changes in weight, PA, and dietary intake are correlated with COVID-19–related perceived barriers and facilitators. The information obtained from participants regarding the reasons for their pandemic-related changes in PA and diet may suggest strategies to encourage healthier behaviors and weight management among people who have been negatively affected by COVID-19 pandemic. In addition, the VL project was implemented by digital program of multiple years, which allowed for minimal changes to programming during the lockdown, and thus this allowed to examine the impact of the COVID-19 independent on behavioral changes and weight loss.

**Conclusion**

Accepted Article

This article is protected by copyright. All rights reserved.

The COVID-19 pandemic was negatively associated with health-compromising eating behavior among the VL program participants, and these participants showed efforts to keep healthy behaviors during lockdown. Future studies are needed to investigate and provide solutions for the physical, behavioral, and mental health problems experienced by people who have been affected by the long-term COVID-19 pandemic.

**Acknowledgement**

This study was supported by the Center for Energy Balance in Cancer Prevention and Survivorship of MD Anderson Cancer Center. We thank Ruth Rechis, PhD, Alise Neff, and Amber Macneish who have worked on the VL project. We also thank Laura L. Russell, Research Medical Library at MD Anderson Cancer Center, for editing this manuscript.

Accepted Article

This article is protected by copyright. All rights reserved.

Accepted Article

## References

1. Nepper MJ, McAtee JR, Chai W. Effect of a Workplace Weight-Loss Program for Overweight and Obese Healthcare Workers. American Journal of Health Promotion. 2020;35(3):352-61. doi: 10.1177/0890117120960393.

2. Earnest CP, Church TS. Evaluation of a Voluntary Worksite Weight Loss Program on Metabolic Syndrome. Metab Syndr Relat Disord. 2015;13(9):406-14. Epub 2015/08/25. doi: 10.1089/met.2015.0075. PubMed PMID: 26302220.

3. Davis J, Clark B, Lewis G, Duncan I. The impact of a worksite weight management program on obesity: a retrospective analysis. Popul Health Manag. 2014;17(5):265-71. Epub 2014/04/17. doi: 10.1089/pop.2013.0108. PubMed PMID: 24735259.

4. Thorndike AN, Healey E, Sonnenberg L, Regan S. Participation and cardiovascular risk reduction in a voluntary worksite nutrition and physical activity program. Prev Med. 2011;52(2):164-6. Epub 2010/12/07. doi: 10.1016/j.ypmed.2010.11.023. PubMed PMID: 21130804; PubMed Central PMCID: PMCPMC3026874.

5. Katz DL, O'Connell M, Yeh MC, Nawaz H, Njike V, Anderson LM, et al. Public health strategies for preventing and controlling overweight and obesity in school and worksite settings: a report on recommendations of the Task Force on Community Preventive Services. MMWR Recomm Rep. 2005;54(Rr-10):1-12. Epub 2005/11/02. PubMed PMID: 16261131.

6. Anderson LM, Quinn TA, Glanz K, Ramirez G, Kahwati LC, Johnson DB, et al. The effectiveness of worksite nutrition and physical activity interventions for controlling employee overweight and obesity: a systematic review. Am J Prev Med. 2009;37(4):340-57. Epub 2009/09/22. doi: 10.1016/j.amepre.2009.07.003. PubMed PMID: 19765507.

This article is protected by copyright. All rights reserved.

7. Benedict MA, Arterburn D. Worksite-based weight loss programs: a systematic review of recent literature. Am J Health Promot. 2008;22(6):408-16. Epub 2008/08/06. doi: 10.4278/ajhp.22.6.408. PubMed PMID: 18677881.

8. Thorndike AN, Sonnenberg L, Healey E, Myint UK, Kvedar JC, Regan S. Prevention of weight gain following a worksite nutrition and exercise program: a randomized controlled trial. Am J Prev Med. 2012;43(1):27-33. Epub 2012/06/19. doi: 10.1016/j.amepre.2012.02.029. PubMed PMID: 22704742; PubMed Central PMCID: PMCPMC3377937.

9. Østbye T, Stroo M, Brouwer RJ, Peterson BL, Eisenstein EL, Fuemmeler BF, et al. Steps to Health employee weight management randomized control trial: short-term follow-up results. J Occup Environ Med. 2015;57(2):188-95. Epub 2015/02/06. doi: 10.1097/jom.0000000000000335. PubMed PMID: 25654520.

10. Gostin LO, Wiley LF. Governmental Public Health Powers During the COVID-19 Pandemic: Stay-at-home Orders, Business Closures, and Travel Restrictions. JAMA. 2020;323(21):2137-8. doi: 10.1001/jama.2020.5460.

11. Thomson M, Spence JC, Raine K, Laing L. The association of television viewing with snacking behavior and body weight of young adults. Am J Health Promot. 2008;22(5):329-35. Epub 2008/06/04. doi: 10.4278/ajhp.22.5.329. PubMed PMID: 18517093.

12. Moynihan AB, van Tilburg WA, Igou ER, Wisman A, Donnelly AE, Mulcaire JB. Eaten up by boredom: consuming food to escape awareness of the bored self. Front Psychol. 2015;6:369. Epub 2015/04/18. doi: 10.3389/fpsyg.2015.00369. PubMed PMID: 25883579; PubMed Central PMCID: PMCPMC4381486.

13. Council IFI. COVID-19 Pandemic Transforms the Way We Shop, Eat and Think About Food, According to IFIC’s 2020 Food & Health Survey 2020 [cited 2022 1/3/2022]. Available from: https://foodinsight.org/wp-content/uploads/2020/06/2020-Food-and-Health-Survey-.pdf.

Accepted Article

This article is protected by copyright. All rights reserved.

14. Lin AL, Vittinghoff E, Olgin JE, Pletcher MJ, Marcus GM. Body Weight Changes During Pandemic-Related Shelter-in-Place in a Longitudinal Cohort Study. JAMA Network Open. 2021;4(3):e212536-e. doi: 10.1001/jamanetworkopen.2021.2536.

15. Flanagan EW, Beyl RA, Fearnbach SN, Altazan AD, Martin CK, Redman LM. The Impact of COVID-19 Stay-At-Home Orders on Health Behaviors in Adults. Obesity (Silver Spring). 2021;29(2):438-45. Epub 2020/10/13. doi: 10.1002/oby.23066. PubMed PMID: 33043562; PubMed Central PMCID: PMCPMC7675243.

16. Almandoz JP, Xie L, Schellinger JN, Mathew MS, Gazda C, Ofori A, et al. Impact of COVID-19 stay-at-home orders on weight-related behaviours among patients with obesity. Clin Obes. 2020;10(5):e12386. Epub 2020/06/10. doi: 10.1111/cob.12386. PubMed PMID: 32515555; PubMed Central PMCID: PMCPMC7300461.

17. Pellegrini M, Ponzo V, Rosato R, Scumaci E, Goitre I, Benso A, et al. Changes in Weight and Nutritional Habits in Adults with Obesity during the "Lockdown" Period Caused by the COVID-19 Virus Emergency. Nutrients. 2020;12(7):2016. doi: 10.3390/nu12072016. PubMed PMID: 32645970.

18. Bullard T, Medcalf A, Rethorst C, Foster GD. Impact of the COVID-19 pandemic on initial weight loss in a digital weight management program: A natural experiment. Obesity (Silver Spring). 2021;29(9):1434-8. Epub 2021/05/20. doi: 10.1002/oby.23233. PubMed PMID: 34009723; PubMed Central PMCID: PMCPMC8456790 International. CR was an employee and shareholder of WW International during the study.

19. Robertson MC, Lee CY, Wu IH-C, Liao Y, Raber M, Parker N, et al. Changes in physical activity associated with the COVID-19 pandemic in individuals with overweight and obesity: an interrupted time series analysis with historical controls. Journal of Behavioral Medicine. 2021. doi: 10.1007/s10865-021-00261-7.

Accepted Article

This article is protected by copyright. All rights reserved.

20. The Diabetes Prevention Program (DPP): description of lifestyle intervention. Diabetes Care. 2002;25(12):2165-71. Epub 2002/11/28. doi: 10.2337/diacare.25.12.2165. PubMed PMID: 12453955; PubMed Central PMCID: PMCPMC1282458.

21. Health Information National Trends Survey (HINTS) [08/23/2022]. Available from: https://hints.cancer.gov/data/survey-instruments.aspx#H5C1.

22. Health of Houston Survey [09/09/2022]. Available from: https://sph.uth.edu/research/centers/ihp/#TID-e1bc0d84-d308-4213-9931-c667967d8c23-3.

23. Aghili SMM, Ebrahimpur M, Arjmand B, Shadman Z, Pejman Sani M, Qorbani M, et al. Obesity in COVID-19 era, implications for mechanisms, comorbidities, and prognosis: a review and meta-analysis. Int J Obes (Lond). 2021;45(5):998-1016. Epub 2021/02/28. doi: 10.1038/s41366-021-00776-8. PubMed PMID: 33637951; PubMed Central PMCID: PMCPMC7909378.

24. Jensen MD, Ryan DH, Apovian CM, Ard JD, Comuzzie AG, Donato KA, et al. 2013 AHA/ACC/TOS guideline for the management of overweight and obesity in adults: a report of the American College of Cardiology/American Heart Association Task Force on Practice Guidelines and The Obesity Society. Circulation. 2014;129(25 Suppl 2):S102-38. Epub 2013/11/14. doi: 10.1161/01.cir.0000437739.71477.ee. PubMed PMID: 24222017; PubMed Central PMCID: PMCPMC5819889.

25. Caldwell AE, Thomas EA, Rynders C, Holliman BD, Perreira C, Ostendorf DM, et al. Improving lifestyle obesity treatment during the COVID-19 pandemic and beyond: New challenges for weight management. Obes Sci Pract. 2021. Epub 2021/09/21. doi: 10.1002/osp4.540. PubMed PMID: 34540266; PubMed Central PMCID: PMCPMC8441901.

26. Ammar A, Brach M, Trabelsi K, Chtourou H, Boukhris O, Masmoudi L, et al. Effects of COVID-19 Home Confinement on Eating Behaviour and Physical Activity: Results of the ECLB-COVID19 International Online Survey. Nutrients. 2020;12(6):1583. doi: 10.3390/nu12061583. PubMed PMID: 32481594.

Accepted Article

This article is protected by copyright. All rights reserved.

27. Dunton GF, Wang SD, Do B, Courtney J. Early effects of the COVID-19 pandemic on physical activity locations and behaviors in adults living in the United States. Prev Med Rep. 2020;20:101241. Epub 2020/11/12. doi: 10.1016/j.pmedr.2020.101241. PubMed PMID: 33173751; PubMed Central PMCID: PMCPMC7644187.

28. Kriaucioniene V, Bagdonaviciene L, Rodríguez-Pérez C, Petkeviciene J. Associations between Changes in Health Behaviours and Body Weight during the COVID-19 Quarantine in Lithuania: The Lithuanian COVIDiet Study. Nutrients. 2020;12(10). Epub 2020/10/18. doi: 10.3390/nu12103119. PubMed PMID: 33065991; PubMed Central PMCID: PMCPMC7599784.

29. Deschasaux-Tanguy M, Druesne-Pecollo N, Esseddik Y, de Edelenyi FS, Allès B, Andreeva VA, et al. Diet and physical activity during the coronavirus disease 2019 (COVID-19) lockdown (March-May 2020): results from the French NutriNet-Santé cohort study. Am J Clin Nutr. 2021;113(4):924-38. Epub 2021/03/07. doi: 10.1093/ajcn/nqaa336. PubMed PMID: 33675635; PubMed Central PMCID: PMCPMC7989637.

30. Minsky NC, Pachter D, Zacay G, Chishlevitz N, Ben-Hamo M, Weiner D, et al. Managing Obesity in Lockdown: Survey of Health Behaviors and Telemedicine. Nutrients. 2021;13(4):1359. doi: 10.3390/nu13041359. PubMed PMID: 33921602.

31. Husain W, Ashkanani F. Does COVID-19 change dietary habits and lifestyle behaviours in Kuwait: a community-based cross-sectional study. Environ Health Prev Med. 2020;25(1):61. Epub 2020/10/14. doi: 10.1186/s12199-020-00901-5. PubMed PMID: 33045996; PubMed Central PMCID: PMCPMC7548533.

32. Scarmozzino F, Visioli F. Covid-19 and the Subsequent Lockdown Modified Dietary Habits of Almost Half the Population in an Italian Sample. Foods. 2020;9(5):675. doi: 10.3390/foods9050675. PubMed PMID: 32466106.

Accepted Article

This article is protected by copyright. All rights reserved.

33. Robinson E, Boyland E, Chisholm A, Harrold J, Maloney NG, Marty L, et al. Obesity, eating behavior and physical activity during COVID-19 lockdown: A study of UK adults. Appetite. 2021;156:104853. Epub 2020/10/11. doi: 10.1016/j.appet.2020.104853. PubMed PMID: 33038479; PubMed Central PMCID: PMCPMC7540284.

34. Abbas AM, Fathy SK, Fawzy AT, Salem AS, Shawky MS. The mutual effects of COVID-19 and obesity. Obes Med. 2020;19:100250-. Epub 2020/05/06. doi: 10.1016/j.obmed.2020.100250. PubMed PMID: 32382684.

Accepted Article

This article is protected by copyright. All rights reserved.

**Figure legends**

Figure 1. Pandemic-related changes in dietary intake and participants' reasons for these changes

Accepted Article

This article is protected by copyright. All rights reserved.

Table 1. Questions for behaviors using items from HINTS

| | Questions |
|---|---|
| **Physical activity** | In a typical week, how many **days** do you do **any physical activity or exercise of at least moderate intensity**, such as brisk walking, bicycling at a regular pace, or swimming at a regular pace? |
| | On days that you do any **physical activity or exercise of at least moderate intensity**, how long do you typically do these activities? (**minutes**) |
| | In a typical week, outside of your job or work around the house, how many **days** do you do leisure-time physical activities specifically designed to **strengthen your muscles**? |
| **Dietary intake** | During the past week, how often did you eat **red meat** such as beef, pork, ham, or sausage? |
| | How many cups of **fruit** (including 100% pure fruit juice) do you eat or drink each day? |
| | How many cups of **vegetables** (including 100% pure vegetable juice) do you eat or drink each day? |
| | How many times did you eat **fast food**? |
| | How many mornings did you have something for **breakfast**? |
| | How often did you drink **sweetened drinks**? |
| | When available, how often do you **use menu information** on calories in deciding what to order? |

HINTS, Health Information National Trends Survey

Accepted Article

This article is protected by copyright. All rights reserved.

Table 2. Participants who completed and dropped out the VL program

| | **Completers (n=266, 70%)** | **Drop-outs (n=114, 30%)** | ***P*-value[a]** |
|---|---|---|---|
| **Mean age, yrs (SD)** | 43.4 (10.3) | 41.9 (9.9) | 0.200 |
| **Race, n (%)** | | | 0.650 |
| Hispanic white | 97 (36) | 43 (38) | |
| Non-Hispanic white | 122 (46) | 57 (50) | |
| Hispanic Black | 1 (1) | 1 (0) | |
| Non-Hispanic Black | 30 (11) | 8 (7) | |
| Asian | 7 (3) | 1 (1) | |
| Other | 9 (3) | 4 (4) | |
| **Female sex, n (%)** | 242 (91) | 98 (86) | 0.145 |
| **Education, n (%)** | | | 0.239 |
| HS/GED or less | 31 (12) | 8 (7) | |
| Tech/vocational degree | 6 (2) | 1 (1) | |
| Some college | 42 (16) | 17 (15) | |
| Bachelor's degree | 84 (32) | 45 (40) | |
| Master's degree | 94 (35) | 37 (32) | |
| Doctoral degree | 9 (3) | 6 (5) | |
| **Marital status, n (%)** | | | 0.914 |
| Single | 52 (20) | 18 (16) | |
| Married/cohabiting | 179 (67) | 79 (69) | |
| Divorced/separated | 35 (13) | 17 (15) | |
| Widowed | 0 (0) | 0 (0) | |
| **BMI category, n (%)** | | | 0.546 |
| Overweight | 66 (25) | 25 (22) | |
| Obese | 200 (75) | 89 (78) | |
| **Mean BMI, kg/m$^2$ (SD)** | 35.4 (6.7) | 36.3 (6.9) | 0.630 |

[a] One-way analysis of variance tests were used for continuous variables (age and BMI) and Pearson's chi-squared tests were used for categorical variables (race, sex, education, marital status, and BMI category).

BMI, body mass index; GED, general educational development; HS, high school; SD, standard deviation

Accepted Article

This article is protected by copyright. All rights reserved.

Table 3. Participants' baseline characteristics

| | Total participants (n=266) (2017-2020) | Non-COVID group (n=173) (2017-2019) | COVID group (n=93) (2019-2020) | *P*-value[a] |
|---|---|---|---|---|
| **Mean age, yrs (SD)** | 43.4 (10.3) | 43.1 (10.3) | 43.9 (10.3) | 0.544 |
| **Race, n (%)** | | | | 0.064 |
| Hispanic white | 97 (36) | 68 (39) | 29 (31) | |
| Non-Hispanic white | 122 (46) | 82 (47) | 40 (43) | |
| Hispanic Black | 1 (1) | 1 (1) | 0 (0) | |
| Non-Hispanic Black | 30 (11) | 14 (8) | 16 (17) | |
| Asian | 7 (3) | 5 (3) | 2 (2) | |
| Other | 9 (3) | 3 (2) | 6 (7) | |
| **Female sex** | 242 (91) | 158 (91) | 84 (90) | 0.785 |
| **Education, n (%)** | | | | 0.584 |
| HS/GED or less | 31 (12) | 23 (13) | 8 (9) | |
| Tech/vocational degree | 6 (2) | 5 (3) | 1 (1) | |
| Some college | 42 (16) | 28 (16) | 14 (15) | |
| Bachelor's degree | 84 (32) | 56 (32) | 28 (30) | |
| Master's degree | 94 (35) | 56 (32) | 38 (41) | |
| Doctoral degree | 9 (3) | 5 (3) | 4 (4) | |
| **Marital status, n (%)** | | | | 0.286 |
| Single | 52 (20) | 35 (20) | 17 (18) | |
| Married/cohabiting | 179 (67) | 116 (67) | 63 (68) | |
| Divorced/separated | 35 (13) | 22 (13) | 13 (14) | |
| Widowed | 0 (0) | 0 (0) | 0 (0) | |
| **BMI category, n (%)** | | | | 0.346 |
| Overweight | 66 (25) | 37 (21) | 29 (31) | |
| Obese | 200 (75) | 136 (79) | 64 (69) | |
| **Mean BMI, $kg/m^2$ (SD)** | 35.4 (6.7) | 35.7 (6.8) | 34.9 (6.6) | 0.078 |

[a] One-way analysis of variance tests were used for continuous variables (age and BMI) and Pearson's chi-squared tests were used for categorical variables (race, sex, education, marital status, and BMI category).

BMI, body mass index; GED, general educational development; HS, high school; SD, standard deviation

Accepted Article

This article is protected by copyright. All rights reserved.

Table 4. Weights, physical activity amounts, and dietary intakes of the non-COVID and COVID groups at baseline and follow-up

| | **Non-COVID (2017-2019) n=173** | | **COVID (2019-2020) n=93** | | **Time** | **Group** | **Time* Group** |
|---|---|---|---|---|---|---|---|
| | **Baseline** | **Follow-up** | **Baseline** | **Follow-up** | ***P*-value** | ***P*-value** | ***P*-value** |
| **Weight** | | | | | | | |
| Mean weight, kg (SD)[a] | 96.2 (19.9) | 93.9 (20.4) | 95.9 (21.6) | 94.6 (21.1) | **<.001** | .903 | .068 |
| 5% weight loss, n (%)[b] | 37 (21) | | 13 (14) | | - | **.048** | - |
| **Physical activity** | | | | | | | |
| Mean total minutes (SD)[a] | 79.8 (83.1) | 128.5 (114.0) | 73.8 (98.0) | 135.3 (185.5) | **<.001** | .494 | .517 |
| MVPA ≥ 150 min/wk, n (%)[c] | 25 (17) | 55 (32) | 14 (15) | 32 (34) | **<.001** | .756 | .573 |
| Strengthening exercise ≥ 2 times/wk, n (%)[c] | 33 (19) | 69 (40) | 18 (19) | 40 (43) | **<.001** | .956 | .747 |
| **Dietary intake, n (%)** | | | | | | | |
| Red meat (≤ 2 days/wk) [c] | 73 (42) | 90 (52) | 40 (43) | 42 (45) | **.019** | .898 | .279 |
| Fruit (≥ 1 cup/day) [c] | 48 (28) | 78 (45) | 30 (32) | 45 (48) | **<.001** | .441 | .783 |
| Vegetables (≥ 3 cups/day) [c] | 31 (18) | 47 (27) | 23 (25) | 27 (29) | **.016** | .189 | .879 |
| Fast food (≤ 2 days/wk) [c] | 95 (55) | 124 (72) | 40 (43) | 72 (77) | **<.001** | .065 | **.008** |
| Menu calorie information used to choose foods (always/often) [c] | 49 (28) | 95 (55) | 34 (37) | 52 (56) | **<.001** | .168 | .263 |
| Breakfast (every day) [c] | 71 (41) | 76 (44) | 33 (35) | 43 (46) | .454 | .376 | .222 |
| Sugar-sweetened beverage consumption (never) [c] | 69 (40) | 63 (36) | 44 (47) | 13 (14) | .378 | .243 | **.016** |

[a] Repeated measure mixed models; [b] Pearson's chi-squared test; [c] Repeated measure logistic regressions

SD, standard deviation; MVPA, moderate to vigorous physical activity

Accepted Article

This article is protected by copyright. All rights reserved.

Accepted Article

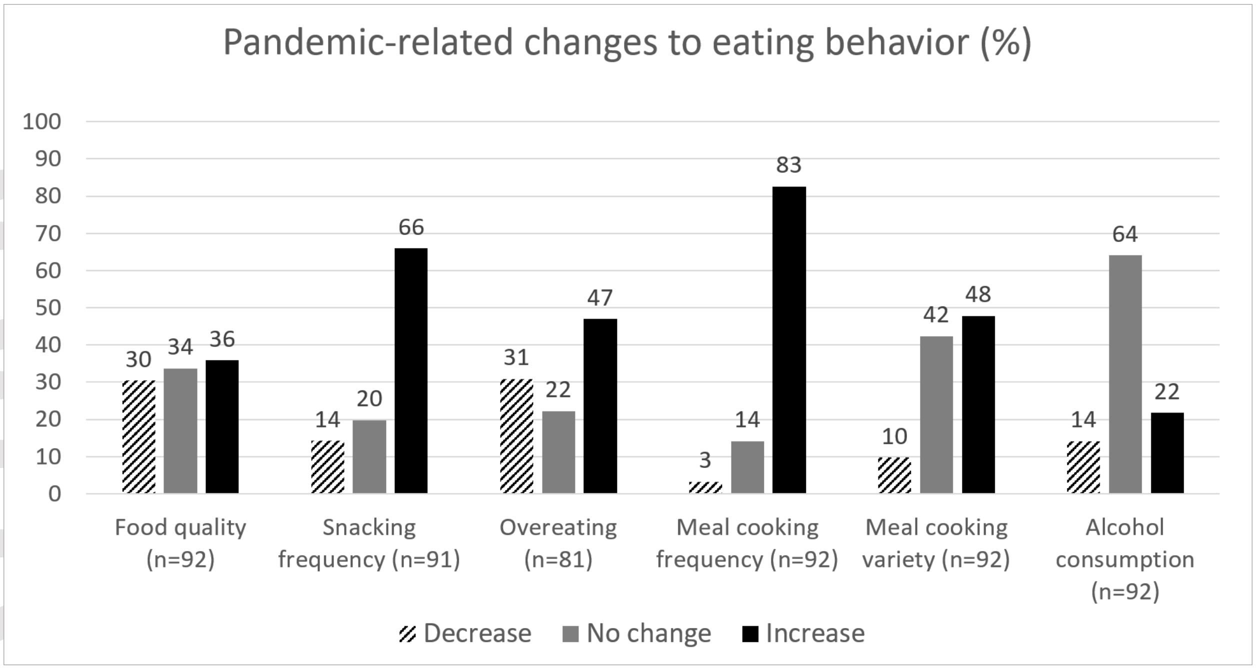

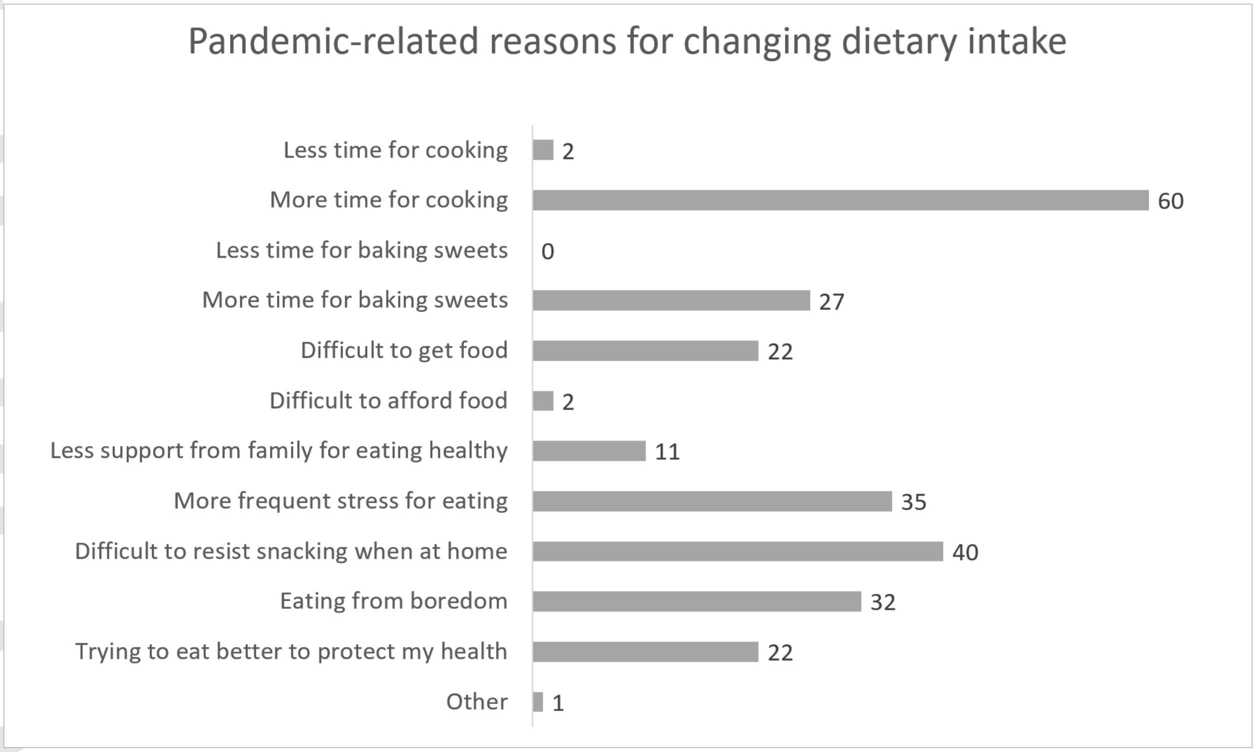

This article is protected by copyright. All rights reserved.

STROBE Statement—Checklist of items that should be included in reports of ***cohort studies***

| | Item No | Recommendation | Page No |
|---|---|---|---|
| **Title and abstract** | 1 | (*a*) Indicate the study's design with a commonly used term in the title or the abstract<br>(*b*) Provide in the abstract an informative and balanced summary of what was done and what was found | 1, 3 |
| **Introduction** | | | |
| Background/rationale | 2 | Explain the scientific background and rationale for the investigation being reported | 4-5 |
| Objectives | 3 | State specific objectives, including any prespecified hypotheses | 5 |
| **Methods** | | | |
| Study design | 4 | Present key elements of study design early in the paper | 6 |
| Setting | 5 | Describe the setting, locations, and relevant dates, including periods of recruitment, exposure, follow-up, and data collection | 6 |
| Participants | 6 | (*a*) Give the eligibility criteria, and the sources and methods of selection of participants. Describe methods of follow-up<br>(*b*) For matched studies, give matching criteria and number of exposed and unexposed | 6 |
| Variables | 7 | Clearly define all outcomes, exposures, predictors, potential confounders, and effect modifiers. Give diagnostic criteria, if applicable | 7 |
| Data sources/ measurement | 8* | For each variable of interest, give sources of data and details of methods of assessment (measurement). Describe comparability of assessment methods if there is more than one group | 7, Table1 |
| Bias | 9 | Describe any efforts to address potential sources of bias | 8 |
| Study size | 10 | Explain how the study size was arrived at | 8 |
| Quantitative variables | 11 | Explain how quantitative variables were handled in the analyses. If applicable, describe which groupings were chosen and why | 8 |
| Statistical methods | 12 | (*a*) Describe all statistical methods, including those used to control for confounding<br>(*b*) Describe any methods used to examine subgroups and interactions<br>(*c*) Explain how missing data were addressed<br>(*d*) If applicable, explain how loss to follow-up was addressed<br>(*e*) Describe any sensitivity analyses | 8 |
| **Results** | | | |
| Participants | 13* | (a) Report numbers of individuals at each stage of study—eg numbers potentially eligible, examined for eligibility, confirmed eligible, included in the study, completing follow-up, and analysed<br>(b) Give reasons for non-participation at each stage<br>(c) Consider use of a flow diagram | 8 |
| Descriptive data | 14* | (a) Give characteristics of study participants (eg demographic, clinical, social) and information on exposures and potential confounders<br>(b) Indicate number of participants with missing data for each variable of interest<br>(c) Summarise follow-up time (eg, average and total amount) | 8, Table2, Table 3 |
| Outcome data | 15* | Report numbers of outcome events or summary measures over time | 8 |

This article is protected by copyright. All rights reserved.

Accepted Article

| | | | |
|---|---|---|---|
| Main results | 16 | (*a*) Give unadjusted estimates and, if applicable, confounder-adjusted estimates and their precision (eg, 95% confidence interval). Make clear which confounders were adjusted for and why they were included<br>(*b*) Report category boundaries when continuous variables were categorized<br>(*c*) If relevant, consider translating estimates of relative risk into absolute risk for a meaningful time period | 9, Table4 Figure1 |
| Other analyses | 17 | Report other analyses done—eg analyses of subgroups and interactions, and sensitivity analyses | NA |
| **Discussion** | | | |
| Key results | 18 | Summarise key results with reference to study objectives | 10 |
| Limitations | 19 | Discuss limitations of the study, taking into account sources of potential bias or imprecision. Discuss both direction and magnitude of any potential bias | 12 |
| Interpretation | 20 | Give a cautious overall interpretation of results considering objectives, limitations, multiplicity of analyses, results from similar studies, and other relevant evidence | 10-12 |
| Generalisability | 21 | Discuss the generalisability (external validity) of the study results | 12 |
| **Other information** | | | |
| Funding | 22 | Give the source of funding and the role of the funders for the present study and, if applicable, for the original study on which the present article is based | 1 |

*Give information separately for exposed and unexposed groups.

**Note:** An Explanation and Elaboration article discusses each checklist item and gives methodological background and published examples of transparent reporting. The STROBE checklist is best used in conjunction with this article (freely available on the Web sites of PLoS Medicine at http://www.plosmedicine.org/, Annals of Internal Medicine at http://www.annals.org/, and Epidemiology at http://www.epidem.com/). Information on the STROBE Initiative is available at http://www.strobe-statement.org.

Accepted Article

This article is protected by copyright. All rights reserved.
